# The EDCMET Project: Metabolic Effects of Endocrine Disruptors

**DOI:** 10.3390/ijms21083021

**Published:** 2020-04-24

**Authors:** Jenni Küblbeck, Taina Vuorio, Jonna Niskanen, Vittorio Fortino, Albert Braeuning, Khaled Abass, Arja Rautio, Jukka Hakkola, Paavo Honkakoski, Anna-Liisa Levonen

**Affiliations:** 1 A.I. Virtanen Institute for Molecular Sciences, University of Eastern Finland, P.O. Box 1627, FI-70210 Kuopio, Finland; jenni.kublbeck@uef.fi (J.K.); taina.vuorio@uef.fi (T.V.); 2 School of Pharmacy, University of Eastern Finland, P.O. Box 1627, FI-70210 Kuopio, Finland; jonna.niskanen@uef.fi (J.N.); paavo.honkakoski@uef.fi (P.H.); 3 Institute of Biomedicine, University of Eastern Finland, P.O. Box 1627, FI-70210 Kuopio, Finland; vittorio.fortino@uef.fi; 4 Department Food Safety, German Federal Institute for Risk Assessment, DE-10589 Berlin, Germany; Albert.Braeuning@bfr.bund.de; 5 Arctic Health, Faculty of Medicine, University of Oulu, P.O. Box 5000, FI-90014 Oulu, Finland; khaled.megahed@oulu.fi (K.A.); arja.rautio@oulu.fi (A.R.); 6 Thule Institute, University of Arctic, University of Oulu, P.O. Box 8000, FI-90014 Oulu, Finland; 7 Research Unit of Biomedicine, Pharmacology and Toxicology, University of Oulu, P.O. Box 5000, FI-90014 Oulu, Finland; jukka.hakkola@oulu.fi; 8 Department of Pharmacotherapy and Experimental Therapeutics, Eshelman School of Pharmacy, University of North Carolina at Chapel Hill, Chapel Hill, NC 27599, USA

**Keywords:** endocrine disruptors (EDs), nuclear receptors (NRs), metabolism, metabolic syndrome, obesity, risk assessment, human health, adverse outcome pathway (AOP), assay validation

## Abstract

Endocrine disruptors (EDs) are defined as chemicals that mimic, block, or interfere with hormones in the body’s endocrine systems and have been associated with a diverse array of health issues. The concept of endocrine disruption has recently been extended to metabolic alterations that may result in diseases, such as obesity, diabetes, and fatty liver disease, and constitute an increasing health concern worldwide. However, while epidemiological and experimental data on the close association of EDs and adverse metabolic effects are mounting, predictive methods and models to evaluate the detailed mechanisms and pathways behind these observed effects are lacking, thus restricting the regulatory risk assessment of EDs. The EDCMET (Metabolic effects of Endocrine Disrupting Chemicals: novel testing METhods and adverse outcome pathways) project brings together systems toxicologists; experimental biologists with a thorough understanding of the molecular mechanisms of metabolic disease and comprehensive in vitro and in vivo methodological skills; and, ultimately, epidemiologists linking environmental exposure to adverse metabolic outcomes. During its 5-year journey, EDCMET aims to identify novel ED mechanisms of action, to generate (pre)validated test methods to assess the metabolic effects of Eds, and to predict emergent adverse biological phenotypes by following the adverse outcome pathway (AOP) paradigm.

## 1. Introduction

The prevalence of metabolic diseases, such as type II diabetes (T2D) and obesity, is rapidly increasing. According to the World Health Organization (WHO), over 460 million people suffer from T2D and 650 million adults are obese, making these diseases major health problems and a significant economic burden worldwide [1,2]. Especially worrying is the continuously growing number of overweight and obese children and adolescents. A cluster of interrelated metabolic risk factors, including elevated blood pressure, abdominal obesity, as well as high plasma glucose, triglyceride, and cholesterol levels, predispose patients to metabolic disorders [3]. This adverse metabolic phenotype further increases the risk of developing T2D [4], non-alcoholic fatty liver disease (NAFLD) [5], as well as cardiovascular complications, such as heart disease and stroke [6]. Genetic background, excessive caloric intake, physical inactivity, sleep deficit, and aging are well-established and -accepted risk factors for metabolic syndrome [3,7]. However, the rapid increase in the prevalence of metabolic disorders during the last two decades as well as the lack of success with traditional interventions have raised an interest in possible additional risk factors [8,9,10,11]. 

One of the major concerns is the human exposure to environmental contaminants, which have been shown to result in adverse health effects [8,9,10,11,12]. Endocrine disruptors (EDs) are exogenous substances or mixtures that alter function(s) of the endocrine system and cause adverse health effects in an organism or its progeny [13,14]. These also include natural compounds, such as hormones and plant toxicants, but mostly they are man-made compounds or their mixtures used as industrial solvents, plastics, plasticizers, flame retardants, fungicides, pesticides, and pharmaceutical agents [14]. EDs can interfere with the endocrine system by numerous mechanisms, such as impeding synthesis, secretion, transport, metabolism, binding, or elimination of natural hormones, or by mimicking hormone action at the level of receptor binding and signal transduction [8,9,10,11,12,13] and may influence the regulation of general homeostasis of the body and contribute to the adverse metabolic phenotypes. Indeed, epidemiological data from humans and experimental data from rodents indicate that exposure to certain EDs, the so-called metabolism disrupting chemicals (MDCs), may predispose patients to different components of metabolic syndrome, T2D, and NAFLD (for reviews, see [15,16,17,18,19,20]). The main targets for MDCs are liver and adipose tissue, where they can provoke, e.g., adipogenesis and fat accumulation [21], insulin resistance [22], and changes in cholesterol and bile acid metabolism [18,19,20]. MDCs have also been shown to regulate nutrient ingestion and metabolism by altering the composition of gut microbiota and intestinal transport. Further, individual host factors may affect susceptibility to the ED-induced harmful effects. The developing fetus is in general much more sensitive to endocrine-like perturbations, and exposure to EDs and MDCs in utero may cause irreversible adverse effects that are only evident much later in life and, thus, have long-term adverse effects on human health [23,24,25]. 

Metabolic diseases are often associated with mitochondrial dysfunction [26]. Therefore, it is not surprising that several MDCs have been shown to modulate processes involved in fatty acid and glucose metabolism including the production and utilization of energy in mitochondria [27,28] (Table 1). MDCs may interfere with these processes either by directly interacting with mitochondrial proteins or secondarily by inducing transcriptional changes in genes coding for these proteins. MDCs can inhibit the production of adenosine triphosphate (ATP), induce the accumulation of reactive oxygen species (ROS), and reduce the mitochondrial membrane potential (MMP). For example, adult exposure to Bisphenol A (BPA) has been shown to decrease the activity of the electron transport chain and lead to increased lipid peroxidation and protein oxidation [29]. Additionally, mitochondrial DNA (mtDNA) is more susceptible to damage than nuclear DNA, and MDCs might increase the number of mtDNA lesions and reduce the mtDNA copy number [28,30,31]. Thus, MDCs may induce mitochondrial dysfunction leading to reduced oxidative capacity, impaired lipid oxidation, and increased oxidative stress and further increase susceptibility to the development of metabolic diseases. 

The effects of hormones are mediated mainly through interactions with their cognate receptors, such as ligand-inducible, transcription-modulating nuclear receptors (NRs) [32,33,34]. In addition to the classical steroid hormone receptors, among the most interesting candidates for ED and MDC effects are the so-called xenobiotic sensors, proteins specialized in sensing the chemical environment and typically involved in the activation of detoxification processes (for reviews, see [33,34,35,36]). Many NRs are well-known targets of several xenobiotics, including EDs and as they have also been shown to be involved in the regulation of cellular energy homeostasis, changes in their activity may underlie many adverse metabolic effects of MDCs [35] (Table 1). EDs may interfere with NR physiology by numerous mechanisms. As small, lipophilic compounds, EDs can directly bind to NR ligand-binding domains and affect cofactor recruitment in a receptor-, ligand-, DNA promoter-, and tissue-specific manner, even at low concentrations. Thus, the potential effects of EDs on downstream gene expression are variable. Furthermore, many examples showing ED interactions with various NRs indicate that one compound may simultaneously interfere with multiple different pathways or multiple compounds may exert synergistic effects [33,37].

Additionally, EDs may exert epigenetic modifications, such as histone modifications, DNA methylation, and the expression of noncoding RNAs [38,39,40]. Epigenetic changes may persist throughout the individual’s lifetime and may also be carried over to next generations. Indeed, many in vivo studies have demonstrated epigenetically-mediated adverse effects, especially to germline cells and to the reproductive system of the offspring after ED exposure. For example, developmental BPA exposure has been shown to modify DNA methylation [41,42] and to alter the expression of different microRNAs (miRNAs) [43]. Mechanistically, EDs regulate the activity of various NRs and transcription factors, thereby also changing the expression of chromatin regulators, such as DNA, histone methyl transferases, and non-coding RNAs (ncRNAs) and may thus interfere with metabolic pathways by inducing epigenetic changes in an NR-dependent manner [44]. 

Despite the emerging evidence linking MDCs to adverse metabolic outcomes, as described above, the current testing tools, including regulatory in vitro and in vivo tests, are not designed to appropriately identify effects related to certain less-studied, endocrine-mediated pathways or health outcomes, in which MDCs may be implicated [45]. New and improved approaches are needed to increase the quality, efficiency, and effectiveness of existing methods to evaluate the effects of MDCs to meet the demanding and evolving regulatory requirements worldwide. Due to the complexity of ED-associated mechanisms and pathways as well as variable environmental exposure, it is of utmost importance to establish more predictive, mechanism-based models for MDC risk assessment.

## 2. EDCMET Objectives

To address the unmet need of validated methods assessing metabolic effects of EDs and to fill in the gaps in knowledge on less-studied ED-induced adverse outcomes, the European Commission has funded eight projects within the Horizon 2020 (H2020) framework. Together, these projects form the European Cluster to Improve Identification of Endocrine Disruptors, (EURION, https://eurion-cluster.eu). The EURION projects are focusing on different aspects of new testing and screening methods to identify Eds, and the cluster will allow the projects to optimize synergies and avoid overlaps as well as share expertise to maximize the impact of the cluster. As one of these projects, the overarching objective of the EDCMET project (www.uef.fi/edcmet) is to develop (pre)validated in silico, in vitro, and in vivo methods assessing the metabolic effects of EDs. EDCMET connects experts in various research fields, including systems toxicologists; experimental biologists with a thorough understanding of the molecular mechanisms of metabolic disease and comprehensive in vitro and in vivo methodological skills; and, ultimately, epidemiologists linking environmental exposure to adverse metabolic outcomes. 

EDCMET aims at producing (pre)validated cell-based and cell-free in vitro functional profiling assays that can specifically identify MDCs and affected metabolic pathways in liver and adipose tissues. EDCMET will also provide an array of in vivo rodent tests suitable for the assessment of the metabolic effects of MDCs through the measurement of physiological functions such as glucose tolerance, dyslipidemia, liver steatosis, and obesity. Further, the project includes efforts in the identification of new tissue and plasma biomarkers, allowing for the prediction of adverse metabolic effects in vivo. Data from human cohort studies will be used to link experimental findings in animals and/or cell cultures to adverse effects in humans. In addition, the project will follow the adverse outcome pathway (AOP) paradigm to identify molecular initiating events (MIEs) and predict the emergent adverse biological phenotypes, as a thorough understanding of the mechanisms leading to adverse metabolic effects in response to EDs exposure is lacking. Thus, EDCMET aims to provide new biological prediction models that link MIEs to metabolic dysfunction (adverse outcomes). A more detailed description of individual project goals and technologies will be presented in the following sections. 

**Figure 1:**
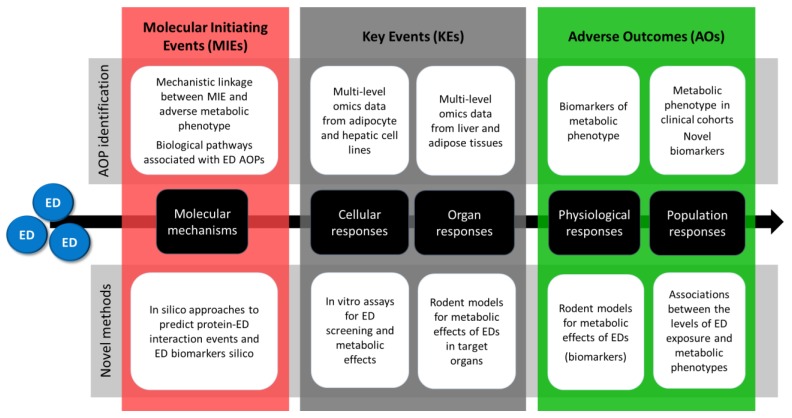
Summary of the overall approach of EDCMET with two parallel, intertwined work streams for the development of novel candidate methods for regulatory use and for the identification of novel adverse outcome pathways (AOPs).

EDCMET will put a strong focus on NRs and the metabolic consequences resulting from their activation or inactivation by foreign compounds. However, novel and currently unidentified mechanisms will also be explored using in vitro and in vivo models utilizing unbiased omics, methods such as genome-wide approaches using next-generation sequencing techniques followed by computational methods to link MIEs to adverse outcomes. This will ultimately provide insights into the mechanisms of action of xenobiotics, which is crucial to the design of appropriate testing methods for metabolic effects exerted via the respective modes of action. EDCMET will therefore not only contribute to test development, but also to the improvement of mechanistic knowledge and AOPs (Figure 1).

## 3. EDCMET Approach

As described, many EDs have been established as MDCs and, as such, have adverse effects on metabolic health [15,16,17,18,19,20,21]. Suspected MDCs from compound classes that have been shown to interact with metabolism-related NRs, have effects on mitochondrial function, or cause adverse metabolic effects, e.g., by inhibiting key enzyme of glucose utilization, are prime candidates for EDCMET studies (Table 1). In order to facilitate the development of reliable and predictive assays, EDCMET will use a core chemical set including MDCs with known molecular mechanisms and large legacy data from several assay types, while ensuring that the compounds cover the relevant chemical space. As more information will be available from both in silico and different experimental approaches, larger sets of compounds will be studied to further test the predictivity of the developed assays and to decipher the mechanisms and pathways behind the metabolic effects of EDs, as indicated in the following sections. 

### 3.1. In Silico Methods to Identify Endocrine Disrupting Effects of EDs

Computational prediction methods aimed at predicting interactions between small molecules and macromolecular targets are widely used in drug development and predictions of toxicity [48]. Molecule-based methods, such as quantitative structure-activity relationship (QSAR), correlate compound structures with biological activity via several molecular descriptors but are often limited in predictivity. Increased availability of structural and functional data has allowed for prediction of more detailed interactions between chemical compounds and potential molecular targets by various molecular docking and molecular dynamics simulation approaches. Due to the need for toxicity assessment of several hundreds of thousands of known and novel synthetic compounds, in silico methods and computational tools are important for reducing costs and the number of animals used for testing. EDCMET will follow two interlinked in silico work streams, which will provide novel or improved and harmonized screening approaches to be used as stand-alone or linkedhybrid approaches. The work streams will be further underpinned through experimental data from in vitro (see Section 3.2) and in vivo (see Section 3.3) approaches as well as the existing literature 

#### 3.1.1. Identification of ED MIEs in Silico

A specific challenge with identification and risk assessment of EDs is related to their varying chemical structure, interactions with numerous cellular targets, and varying effects on different physiological processes [49,50]. Risk assessment requires both the identification of critical MIEs that underpin the adverse outcome and the ability to classify chemicals that can trigger these MIEs. Cutting-edge computational approaches will be used to undertake a high proteome screen and to identify and build a database of potential ED-interacting proteins. The database will comprise all proteins for which a robust 3D structure is available or can be generated by homology modelling (e.g., MODELLER). Various molecular modelling approaches will be used to explore potential interactions between known EDs and proteins. The established and experimentally validated protein–ED combinations will be used as a training set to evaluate different molecular docking approaches (e.g., normal high throughput virtual screening (HTVS), Molecular Mechanics/Generalized Born Surface Area (MM-GBSA) corrections, induced fit) and molecular dynamics simulations to further enable proteome-wide prediction of protein–ED interactions and to determine the optimal and computationally least-demanding approaches for different ED targets, by also using the data obtained from in vitro and in vivo screening of the ED compounds (see Section 3.2 and Section 3.3). The obtained data will further act as a focus for exploring the link between MIEs and adverse outcomes. 

#### 3.1.2. Using Toxicogenomic Data to Model the Mechanistic Linkage between MIE and Adverse Metabolic Phenotype of EDs

The field of toxicogenomics aims to use molecular profiling in order to establish the quantitative relationships between chemical exposure and effects on biological pathways [51]. In particular, gene expression profiles can indicate altered gene expression after toxicant exposure and lead to essential biological pathways involved in toxicity response [52]. These pathways can provide insight into the mode of action of EDs [53] and allow for the characterization of the overall biological phenotype emerging from a chemical exposure. Further, large amounts of toxicogenomic data are now available in the public domain, enabling data re-use and, consequently, the possibility to more robustly model the biological response of a compound treatment as well as to identify the toxicity-related biomarkers.

Thus, we aim to collect and analyze publicly available gene expression data describing the effects of compounds in rat livers (in vivo and in vitro), human hepatocytes, and a human liver cancer cell line. Specifically, DrugMatrix [54], Open TG-GATEs [55], and the Library of Integrated Network-based Cellular Signatures L1000 dataset (LINCS) [56] will be used for the identification of putative molecular mechanisms linking ED MIEs to adverse outcomes. Additionally, the Comparative Toxicogenomics Database [57] will be used to identify the ED MIEs and to provide compound–gene–phenotype associations. Gene expression data, biological annotations, and manually-curated associations between chemicals, genes, phenotypes, and diseases will be fed into data-mining and machine-learning algorithms to characterize the molecular fingerprinting of ED exposures, identify biomarkers, and build classifiers for rapid screening of various ED-mediated effects in vivo.

### 3.2. In Vitro and Omics Methods to Assess Metabolic Effects of EDs

In vitro and omics methods will be utilized to develop cell-free and cell-based profiling assays to identify biochemical and cellular responses to EDs. Further, our aim is to identify ED-affected metabolic pathways and potentially find novel AOPs leading to metabolic disorders that can be applied in future MDC screenings [58]. To fill the gaps in the knowledge of ED-linked health hazards [59] with a widening spectrum of chemical exposures, it is paramount to discover, optimize, and validate high-throughput screening assays for EDs. As described earlier, our primary focus will be on NRs that are involved in the regulation of hepatic metabolism and associated with potential MDCs (see Section 3 and Table 1), according to the existing literature e.g., [60,61] 

First, reference and potential MDCs will be profiled for direct interactions with NRs by applying the Microarray Assay for Real-time Coregulator-Nuclear receptor Interaction (MARCoNI) platform [62]. The MARCoNI method produces a heat map of various coregulator peptides recruited by the test chemicals to the specific NR ligand-binding domain. Second, cell-based reporter gene assays will be optimized with known NR-activating reference compounds. After the initial optimization and pre-validation of these assays [63,64], potential MDCs will be screened for their potency. These compounds have been preselected by using the in silico screening results (see also Section 3 and Section 3.1) and knowledge from prior studies, e.g., [18,65,66,67]. This information will be cross-referenced to the data available in public EU sources and ToxCast [68].

Hepatic and adipose cells will then be exposed to high-ranking MDCs and reference chemicals and subjected to cell-based functional metabolic assays. These include functional assays such as oxygen consumption, extracellular acidification, and lipid and lipoprotein analyses, to visualize the effects at the cellular level. Similarly, RNA and protein expression will be investigated in the cell models exposed to vehicle, reference compounds, and the highest-ranking MDCs. Novel targets that do not act via NR modulation will also be identified based on expression data and gene ontologies. 

Finally, data from the different assays will then be compiled, and the respective assay performances will be evaluated. A ranking list for the assays will be produced and the best-performing assays will be validated with a subset of MDCs. The list of potential MDCs will be continuously pruned by the feedback from the rodent in vivo studies (see Section 3.3) and epidemiological data together with information on the human exposure levels (see Section 3.4), as described above (see Section 3).

### 3.3. In Vivo Models for the Assessment of Metabolic Effects of EDs

Endocrine disruption typically affects processes involving multiple organs and tissues at the systemic level. Therefore, the metabolic disruption can ultimately be detected only in the whole organism or other complex systems. Although the ultimate goal for toxicity testing is the application of non-animal testing methods, it is unlikely that animal testing could be fully replaced in the near future. 

Studies on experimental animals, especially in rodents, are among the most common ways to identify harmful metabolic effects of chemicals, and the scientific literature frequently reports such observations (for reviews see [20,69]). However, metabolic tests have not been incorporated to the current in vivo toxicity testing regimes and there are no established, standardized guidelines for performing these tests for the purpose of detecting metabolic disruption by MDCs. In the EDCMET project we aim to develop such guidelines. Thus, we do not plan to develop news tests requiring new animals, but instead aim to add new endpoints for the current, existing testing methods. As described earlier, one major goal of the EDCMET project is to develop in vitro and in silico methods for the detection of metabolism disruption (see Section 3.1 and Section 3.2). To support this development, we will use in vivo experiments to provide a platform for testing the predictivity by these novel or improved methods. 

Susceptibility to metabolic disturbances may be modified by many individual factors. For example, an existing metabolic disease may increase the risk for ED-induced metabolic dysfunction. Therefore, we aim to investigate if obese subjects could be especially vulnerable to the metabolic effects of EDs. We aim to provide novel understanding of the mechanisms mediating the harmful metabolic effects of chemicals. This work involves use of transgenic mouse models. In the context of the AOP concept, the mode of action data should help to identify the MIEs beginning the sequence of events resulting in toxicity, the key events (KE) involved, and the final adverse outcome. In conjunction with that, we will investigate the possibility of identifying new potential plasma and/or tissue biomarkers for metabolic disruption utilizing unbiased omics techniques.

### 3.4. Population-Based Assessment of Exposure and ED-Related Metabolic Effects

In silico, in vitro, or animal models have only a minor relevance unless they are coupled with exposure data that links the levels of chemicals with metabolic endpoints in humans. The objective of the population-based assessment is to assess the levels of exposure to EDs in select cohorts in Finland, Norway, and Spain with different exposure patterns, and to associate pollutant exposure with metabolic anthropometric data as well as genetic susceptibility. A cost-effective nuclear magnetic resonance (NMR) metabolomics to interrogate metabolic parameters within existing cohorts will be employed to potentially identify novel biomarkers for metabolic effects of EDs. EDCMET has access to several relevant cohorts (Table 2) that will be used to correlate the levels of exposure to metabolic endpoints in humans.

In the first phase of the project, the pre-validated methods will be used to assess the concentrations of several MDCs in selected cohorts from the collected samples or from ongoing sampling at birth of newborns and mothers, in children, or adults. Low birth weight, short anogenital distance, and incidence of cryptorchidism and hypospadias have been associated with the effects of EDs in newborns, and subsequently with excess weight, high body mass index (BMI), and diabetes in adults. As described above, EDs may lead to a variety of health outcomes, such as obesity, diabetes mellitus, metabolic syndrome, alterations of female and male reproductive function (including infertility), behavioral and developmental disorders, and hormone-sensitive cancers. 

Identification of associations between human exposure and endocrine effects is not easy given the diversity of the environmental exposure. Therefore, the search for novel biomarkers for future use to estimate the exposure of MDCs in human populations is crucial. The level of exposure will be associated with the metabolism-related parameters collected from cohorts. Genotyping data will be used to assess the genetic susceptibility to adverse metabolic effects of EDs that can be used to identify genetically predisposed individuals. Metabolism-related parameters have been measured in a large proportion of NFBC1966 and NFBC1986 cohorts using the serum NMR platform [70,71]. The high-throughput NMR spectroscopy platform is a cost-effective and well-validated method for simultaneous quantification of 230 metabolic features that represent a broad molecular signature of the systemic metabolite profile. The quantified features are related to lipoprotein subclasses, serum proteins, lipids, fatty acids, and abundant metabolites such as glycolysis substrates, amino acids, ketone bodies, and other small molecules.

Knowledge gained from previous steps is crucial for the development of a study protocol suitable for detecting and following the health outcomes after exposure to ED-related compounds during pregnancy or later in life. Additionally, new MDCs identified from in silico screening for ED–protein interaction (see Section 3 and Section 3.1) will be measured using appropriate chromatographic and mass spectrometry methodologies from the cohort samples. This will allow for the experimental check of the in silico MDC results by analyzing the new compounds in human fluids and studying the metabolic dysfunctions in the cohorts. Human health effects associated with exposure to environmental contaminants could be estimated through several approaches. Blood reference values are employed as toxicological cut-off points for the evaluation of potential health outcomes, but this is not the case with EDs. We aim to incorporate relevant information for specific MDCs from other studies (e.g., in silico methods, in vitro cell culture, and in vivo models) to complement the estimation of human exposure and health risks to a specific MDCs based on actual population exposure scenarios.

## 4. Expected Outcomes, Impact, and Future Perspectives

The current testing methods do not appropriately identify effects related to less-studied endocrine-mediated pathways and health outcomes but are more focused on tests and endpoints for chemicals interfering with hormones involved in reproductive functions. The developed new methods are expected to identify novel disrupted pathways in human liver and adipose tissues and to increase human relevancy in testing and risk assessment. The developed in vitro testing tools and the AOP approach are an attempt to shift from the use of laboratory animals towards the use of more human-like in vitro and computational techniques. Reducing the use of laboratory animals has a significant effect on the time and cost required for assessing toxic potential of chemicals, which makes the risk assessment economically more advantageous. Metabolic diseases are very prevalent and represent a high cost for healthcare systems in the EU countries. Our project will enhance the understanding of metabolic effects of EDs and may provide novel biomarkers for metabolic ED effects in humans. Providing better tools for the assessment of MDC effects will ultimately increase chemical safety, thereby positively impacting environmental health.

The EDCMET project is expected to produce several in silico and in vitro (pre-validated) methods for the detection of suspected MDCs. These assays will then be forwarded for thorough validation and automation, e.g., by the Joint Research Centre of the European Union, and potentially for acceptance by the international community via the Organisation for Economic Co-operation and Development (OECD). We also aspire to complement the existing metabolic assays by omics-based techniques. In addition to yielding genome-wide data, the omics analysis of cellular and tissue samples will also generate pipeline procedures that can be used directly or after slight modification by future toxicological studies.

EDCMET will gain strength from collaboration with other EURION projects that communicate best practices, in, e.g., data management and data sharing, and discuss common methodological approaches or problems in cross-cutting working groups within EURION and from EURION joint meetings where representatives of the regulatory agencies also participate. The interactions and collaborations with OBERON and GOLIATH projects that tackle the same metabolic disorders but use complementary methodologies will be especially fruitful. The use of commonly agreed EDs across many if not all assay platforms within the EURION projects should lend further validity to the findings.

## Figures and Tables

**Table 1:** Metabolism disrupting chemicals (MDCs), their nuclear receptors (NRs) and other transcription factor targets, epigenetic modifications, and mitochondrial effects [27,33,34,35,36,37,38,39,46,47].

ED Chemical Group	Example	Energy Metabolism Cholesterol and Bile Acid Metabolism							Partner for Other NRs	Classical ED Targets		Epigenetic Modifications	Mitochondrial Effects	
		CAR PXR	ERR	GR	TR VDR	PPARs	LXR FXR	AhR	RXR	ER	AR	DNA Methylation Histone Modifications ncRNAs	Bioenergetics	Oxidative Stress
Alkylphenols and derivatives	Nonylphenol	x	x							x	x	x	x	
Antifoulants	Tributyltin			x		x			x				x	
Bisphenols	BPA	x	x	x		x				x	x	x	x	x
Flame retardants	PBDE	x	x			x								
Fungicides	Conazoles, Vinclozolin	x		x	x	x		x			x	x		
Herbicides	Atrazine												x	
Parabens	Butylparaben									x	x	x		
POPs	PCBs, DDT	x	x		x			x		x		x		
Pesticides and insecticides	Thiacloprid, Cypermethrin	x	x		x		x		x	x	x		x	x
Phthalates	DEHP	x			x	x				x		x		
Polyfluorinated chemicals	PFOS	x				x						x		x

x indicates known effects of these compounds on NR activity or epigenetic modifications or mitochondrial function. BPA: Bisphenol A, PBDE: Polybrominated diphenyl ethers, POP: Persistent organic pollutants, PCB: Polychlorinated biphenyl, DDT: Dichlorodiphenyltrichloroethane, DEHP: Bis(2-ethylhexyl) phthalate, PFOS: Perfluorooctanoic acid, CAR: Constitutive androstane receptor, ERR: Estrogen-related receptor, GR: Glucocorticoid receptor, TR: Thyroid hormone receptor, VDR: Vitamin D receptor, PPAR: Peroxisome proliferator-activated receptor, LXR: Liver X receptor, FXR: Farnesoid X receptor, AhR: Aryl hydrocarbon receptor, RXR: Retinoid X receptor, ER: Estrogen receptor, AR: Androgen receptor, ncRNA: non-coding RNA.

**Table 2:** Overview of epidemiological cohorts connecting environmental exposure to metabolic disorders in the EDCMET project.

Country	Cohort *	Recruitment Period	Number of Participants	Description
Finland	KuBiCo	2012–2018 Follow-up: 6 years	5000 women/child pairs	Prospective collection, child-mother cohort study. KuBiCo focused on effects of different stress factors (medicines, nutrition, lifestyle factors, and environmental aspects) during pregnancy on health status of mother and child
Norway	MISA	2007–2009 2014–2015	515 50 555 women	Cross-sectional study with longitudinal aspects aimed to assess exposure experienced by pregnant women and their newborns to a suite of environmental pollutants
Norway	MISA2	2017–2018	700 women	Cross-sectional study with longitudinal aspects aimed to assess exposure experienced by pregnant women and their newborns to a suite of environmental pollutants
Spain	INMA	1997–1998 2000–2008	102 3794 3896 women/child pairs	Prospective population-based cohort concerned with the effects of pre- and postnatal exposures on growth, health, and development from early fetal life until adolescence
Finland	NFBC1966	1966; Follow-up: 6–12 m, 14–16 y, 31 y and 46 y	12,068 mothers and 12,231 children	Longitudinal and prospective birth cohort of women and offspring (prospective data collection from maternity cards since 16th gestational week)
Finland	NFBC1986	07/1985–06/1986; Follow-up: 6–12 m, 7–8 y, 14–16 y and 31 y (2019–2020)	9362 mothers and 9479 children	Prospective data collection from 10th gestational week

* KuBiCo: Kuopio Birth Cohort; MISA: Northern Norway mother-and-child contaminant cohort study; INMA: INfancia y Medio Ambiente (Environment and Childhood); NFBC: The Northern Finland Birth Cohort.

## Abbreviations

AhR	Aryl hydrocarbon receptor
AOP	Adverse outcome pathway
AR	Androgen receptor
ATP	Adenosine triphosphate
BMI	Body mass index
BPA	Bisphenol A
CAR	Constitutive androstane receptor
DDT	Dichlorodiphenyltrichloroethane
DEHP	Bis(2-ethylhexyl) phthalate
ED	Endocrine disruptor
ER	Estrogen receptor
ERR	Estrogen-related receptor
FXR	Farnesoid X receptor
GR	Glucocorticoid receptor
H2020	Horizon 2020 framework programme
HTVS	High throughput virtual screening
INMA	INfancia y Medio Ambiente (Environment and Childhood project)
KE	Key event
KuBiCo	Kuopio Birth Cohort
LINCS	Library of Integrated Network-Based Cellular Signatures
LXR	Liver X receptor
MARCoNI	Microarray Assay for Real-time Coregulator-Nuclear receptor Interaction
MDC	Metabolism disrupting chemical
MIE	Molecular initiating event
MISA	Miljøgifter i Svangerskapet og i Ammeperioden (Northern Norway Mother-and-Child contaminant Cohort study)
MM_GBSA	Molecular mechanics/Generalized Born Surface Area
MMP	Mitochondrial membrane potential
NAFLD	Non-alcoholic fatty liver disease
NFBC	The Northern Finland Birth Cohort
NMR	Nuclear magnetic resonance
NR	Nuclear receptor
OECD	Organisation for Economic Co-operation and Development
PBDE	Polybrominated diphenyl ether
PCB	Polychlorinated biphenyl
PFOS	Perfluorooctanesulfonic acid
POP	Persistent organic pollutants
PPAR	Peroxisome proliferator-activated receptor
QSAR	Quantitative structure-activity relationship
RXR	Retinoid X receptor
T2D	Type II diabetes
TR	Thyroid hormone receptor
VDR	Vitamin D receptor
WHO	World Health Organization
